# Disease, Dignity and Palliative Care

**DOI:** 10.4103/0973-1075.68400

**Published:** 2010

**Authors:** MR Rajagopal

**Affiliations:** Chairman, Pallium India and Director, Trivandrum Institute of Palliative Sciences, PJRRA 65, Pothujanam Road, Kumarapuram, Thiruvananthapuram - 695 011, Kerala, India. E-mail: mrraj47@gmail.com

I once had the privilege of treating a remarkable person, a senior Government official with advanced cancer. She was suffering from terrible pain, had a colostomy, and appeared generally miserable. I asked her a routine question, “What bothers you most?” Her face came alive for a moment, but then she shook her head and said, “I guess you had better attend to my pain first”. Later on during one of our conversations, she brought up the subject. “You know”, she said, “I have been thinking about your question. I think what I minded most were the insults."

She had felt insulted when, just because she was ill, anyone could call her by her first name. She was no longer “Madam” or “Mrs. So and So”; even the youngest doctor or nurse, many years her junior, would address her by name. What right did they have to do this, she asked with a spark of anger. “It is not my ego”, she said, “It was just simply improper. You do not address your seniors by their first name in our culture. It is not done in your home, office or on the street, so why should it happen as soon as you are ill?”

That was not all. They made her discard her clothes and instead wear a gown which left the back of her chest practically naked and left her very uncomfortable. Why should they, without any reasonable explanation whatsoever, attempt to convert her from a person to a container of disease clumsily wrapped in a printed gown? She did bring it up with a senior consultant, who she said insulted her further with an insensitive giggle as if to say, “You are silly!”, touched her on the shoulder patronizingly and asked her not to mind these minor things, but “to behave like a grown up”. These apparently *minor* things actually seemed to matter to her more than the cancer because while the cancer was inevitable this other suffering was unnecessary and hence unacceptable.

What she perhaps did not know was the extent to which human suffering could be compounded by loss of dignity in the face of poverty. They may have very little of worldly possessions, but the one thing that the poor in our country proudly live with, is their dignity. Adversities are a part of their life, but disease removes them to a white-coated disinfectant-scented alien place where they no longer have an identity. They have no protection at all from insults. Anyone at all, from the security guard at the gate to the doctor, is free to abuse them with words and gestures.

The very word “patient” tends to favor stigmatization. “Patient” comes from the Latin word *patiens*, meaning to endure, bear, or suffer, and refers to “an acquired vulnerability and dependency imposed by changing health circum-stances”.[1] The system seems to say, if you are ill you are supposed to *endure, bear or suffer*; so why do you complain if we are arrogant and insulting?

Palliative care came like a breath of fresh air amidst this disease-oriented medical nightmare. By concentrating on quality of life, palliative care turned the focus of treatment from *disease* to the *person* and also to his family.[2] It brought attention to the various factors necessary to help the person with the disease to live until he dies. Understandably, perhaps, the physical aspects of care receive more attention than others. Psychological, social and spiritual issues (in that order) seem to get less attention in actual practice. Even when some exploration does happen, the patient is unlikely to come out in the open and say, “your system insults me”. The unrecognized deprivation of dignity keeps wounds raw and prevents healing.

Restoration of dignity, hence, is essential for psychological, social and spiritual well-being.

The following are essential components of the process.

## RESPECT

Of the major factors needed to maintain dignity in the face of disease, the oft-forgotten one is respect. The person with disease is weak and vulnerable; the professional is in a position of power. Abuse of this power is usually an unconscious process, making the professional behave in ways in which the vulnerable person feels insulted. It may manifest as overt disrespect or it may be more subtle - like condescension, being judgmental or even as inappropriate humor. And any abuse of power has a particularly huge impact in this context because the person with the disease is totally *unarmed*; he has no strength to react to insults. Strangely, the professional is usually unaware that he is abusing power. Unless the issue is discussed, he has no opportunity to change. It is also important to make “respect” an essential point to be made forcefully during palliative care teaching. Students and trainees learn attitudes by role modeling more than in any other way, and disrespectful behavior is a likely unconscious acquisition that needs to be undone by role plays and practice. It also helps if the professional/trainee gets an opportunity to think and realize that the person with disease is likely to be superior to him in some way or other.

## EMPATHY

A starting point would be the question, “How would I feel if I were in his position?” But it is only a starting point. It is important to recognize that not every human mind works in the same way. Everyone has a right to be different and we should consciously avoid the trap of assuming how *he* feels. Nevertheless, this question to oneself is still important.

## RELIEF FROM SYMPTOMS

Uncontrollable pain or other symptoms have such an impact on the psyche that the family may have to watch the loved person change into a self-centered individual, always irritable and demanding attention, perhaps oblivious to the suffering of others.

## COMPANIONSHIP AND ENCOURAGING LIFE REVIEW

Anatole Broyard’s (former editor of New York Times Book Review) description of his experience with prostate cancer includes the following lamentation, “I just wish he (*the physician*) would give me his whole mind just once, be bonded with me for a brief space, survey my soul as well as my flesh, to get at my illness, for each man is ill in his own way”.[3] It helps when someone sits down beside the person and seems to be ready to listen. That process of being there itself acknowledges the person as important. A statement from such a *companion* like, “Life is not very easy for you at the moment, is it?” may then be enough to open the emotional floodgates and to bring out pent up feelings and emotions. Listening to the descriptions of the person’s struggles with life, the successes and the failures acknowledges that life as important.

Life review often helps the person to explore his own mind and to find an answer to questions like “What was the meaning of my life?” Even if that is not achieved, he may at least be left feeling, “Well, my story, my life, was important enough to interest someone!”

Thus, we see that respect, empathy, symptom control, companionship and encouraging life review are essential ingredients in the process of maintaining or restoring dignity. Without them, palliative care would be incomplete. To achieve all of them for many would be an impossible task for the isolated professional. Hence team work is essential.

Another question arises from this. Why should all this be limited to palliative care? Why should people go through pain, humiliation and destruction of dignity almost all through the process of illness till they get respect, empathy and the works only in the last few days or weeks of life?

All that palliative care stands for should be integral to the whole of health care.
